# Trabecular Bone Score and Bone Mineral Density in Postmenopausal Women with Morbid Obesity—A Clinical Paradox

**DOI:** 10.3390/medsci9040069

**Published:** 2021-11-09

**Authors:** Antresa Jose, Kripa Elizabeth Cherian, Munaf Babajan Nandyal, Stephen A. Jiwanmall, Dheeraj Kattula, Thomas V. Paul, Nitin Kapoor

**Affiliations:** 1 Department of Endocrinology, Diabetes and Metabolism, Christian Medical College, Vellore 632004, India; antresa.jose.pg@cmcvellore.ac.in (A.J.); kripaec@cmcvellore.ac.in (K.E.C.); tpaul@cmcvellore.ac.in (T.V.P.); 2 Department of Psychiatry, Christian Medical College, Vellore 632004, India; munaf@cmcvellore.ac.in (M.B.N.); stephen76@cmcvellore.ac.in (S.A.J.); dheeraj.kattula@cmcvellore.ac.in (D.K.)

**Keywords:** morbid obesity, South Asian phenotype, bone obesity paradox, osteoporosis

## Abstract

Obesity has long been considered to have a protective effect on bone, but specific complications in those with morbid obesity are known to have a detrimental impact on bone architecture. We aimed to study the bone microarchitecture (TBS—trabecular bone score) and bone mineral density (BMD) in postmenopausal women with morbid obesity compared to obese and non-obese age-matched women. Eighty-five consecutive postmenopausal women with morbid obesity (body mass index (BMI) ≥ 35 kg/m^2^) were enrolled and compared to age-matched obese (*n* = 80) and non-obese postmenopausal controls (*n* = 85). The BMD and TBS were assessed in all subjects using a Hologic-QDR 4500-W Discovery-A DXA scanner. The mean BMD (gm/cm^2^) at the femoral neck in women with morbid obesity was found to be significantly lower as compared to the age-matched postmenopausal obese controls (0.723 versus 0.762, *p*-value = 0.002). The BMD at the lumbar spine and hip showed similar trends but were not statistically significant. The bone microarchitecture was found to be significantly lower in those with morbid obesity (1.205) as compared to the other two groups (obesity 1.244; non-obese 1.228) (*p* < 0.013). Though obesity was associated with a better bone density and bone microarchitecture in postmenopausal women, a paradoxical lower value was seen in those with morbid obesity.

## 1. Introduction

Obesity and osteoporosis are two rapidly emerging public health problems [1,2]. Conventionally, increased body fat is believed to restrain bone loss and prevent osteoporotic fragility fractures [3]. Several previously published studies have shown a linear negative correlation between the BMI and fractures [4,5,6,7,8]. However, there is emerging literature to suggest that some individuals with obesity may be prone to have certain peripheral fractures [9,10]. This concern may be compounded in individuals who have morbid obesity, as they bear a much larger weight on peripheral bones. Moreover, they have nutritional restrictions, limited mobility, poor sunlight exposure, and higher inflammatory cytokines, which may have a detrimental impact on the bone mineral density [11].

There is a rising prevalence of obesity in the Indian population [12,13]. The INDIAB study in India (first phase) showed a prevalence of generalized obesity in the range of 1.6–31.3% in various states of India. The prevalence of abdominal obesity, defined as waist circumferences ≥90cm in men and ≥80 cm in women, was in the range of 18.9–36.1% [14]. Women have shown to have a higher prevalence of obesity than men, which was confirmed in this study, as well as the national family health survey-3 data [15]. In addition, recent data from a Vellore birth cohort suggested a nearly 100 percent increase in the prevalence of obesity in women over the last 10 years.

Moreover, previously published literature suggested that South Asians have a poor peak bone mass, poor dietary calcium intake, and an earlier age of menopause [16]. This is further compounded by the Indian metabolic phenotype having a unique body phenotype with large amounts of bioactive visceral adipose tissue, especially in those with morbid obesity [12,13,17]. To date, there has been no published literature on the bone health of morbidly obese postmenopausal women who descend from the Asian–Indian lineage.

In addition to BMD, microarchitectural alterations can significantly contribute to the fracture risk. The trabecular bone score (TBS) is a densitometric tool that evaluates pixel gray-level variations in lumbar spine DXA images, providing an indirect measure of the bone microarchitecture [18,19,20,21,22,23,24]. There is a paucity of literature on bone microarchitecture in women with morbid obesity and no available data from the South Asian region.

In this study, we aim to determine the bone mineral density (BMD) in postmenopausal women with morbid obesity and compare it to obese and normal weight age-matched controls.

## 2. Material and Methods

This was a cross-sectional single center study in which a total of 250 postmenopausal women were recruited after written informed consent. The study subjects were categorized based on body mass index. Eighty-five subjects with morbid obesity (BMI ≥ 35 kg/m^2^) were recruited from the multidisciplinary obesity clinic in the hospital, and they were compared to 80 subjects with obesity (BMI ≥ 25–35 kg/m^2^) and 85 non-obese women (BMI ≤ 25 kg/m^2^) recruited from the community. The BMI cut-off points for obesity were defined as per the Asia-Pacific guidelines by the WHO, which suggest lower thresholds for obesity to identify the higher at-risk individuals at a lower BMI [25,26,27,28,29,30,31,32]. This study was approved by the Institutional Review Board (IRB Min no. 10146). The subjects were recruited using simple random sampling over a period of one year from April 2018 to March 2019. All subjects provided written informed consent at the time of recruitment to the study. Postmenopausal women who were on treatment for osteoporosis, those with chronic kidney disease stages 4 and 5, those with secondary osteoporosis, and those taking medications (except calcium and vitamin D supplements) known to affect bone health were excluded from the study.

The bone mineral density (BMD) was assessed at the femoral neck (FN) and lumbar spine (LS) in all subjects using a dual-energy X-ray absorptiometry (DXA) scanner—Hologic QDR 4500 (MedPlus Equipment Service, Madison Heights, MI, USA). The precision was 2 percent at the lumbar spine and 5 percent at the femoral neck. The QC of the DXA machine was done using a specified phantom, and the precision was calculated using 15 patients, 3 scans per patient. WHO classification was used for categorization of the BMD. A T-score of −1.0 or above was diagnosed as normal, a T-score between −1.0 and −2.5 was considered as osteopenia, and a T-score of less than −2.5 was categorized as osteoporosis [33]. The TBS was assessed by the same machine, which provided a noninvasive method that evaluated pixel gray-level variations in the lumbar spine DXA image and helped in assessing the microarchitecture of the bone. Blood biochemical data were collected, which included albumin-corrected calcium (N: 8.3–10.4 mg/dL), phosphate (N: 2.5–5 mg/dL), 25-hydroxy Vitamin D (N: 30–75 ng/mL), and creatinine (N: 0.6–1.2 mg/dL). 

Based on a previous study, with a difference in the mean BMD (femoral neck) of 0.063 g/cm^2^ and difference in the SD of 0.020, keeping a power of 80 percent and alpha error of 5 percent, the sample size in each group was calculated to be 80 cases using electronic software nmaster [9]. Continuous variables were expressed as the mean and SD, and the categorical variables were expressed as the frequencies and percentages. The differences in the means of the continuous variables in the two groups were compared using a Student’s *t*-test for normally distributed parameters. One-way Analysis of Variance (ANOVA) was used to calculate the significance among 3 groups. For all comparison, a two-tailed *p*-value of <0.05 was considered statistically significant. Data were analyzed using SPSS v 24.0 (SPSS IBM Corp, Armonk, NY, USA).

## 3. Results

The baseline characteristics are summarized in Table 1. The mean age and bone biochemistry were similar in the three groups. There were 33% of individuals with hypertension, 34% with prediabetes/type 2 diabetes mellitus, and 54% with dyslipidemia in the morbidly obese group. The number of individuals with prediabetes/diabetes was 28% in the obese group and 17% in the non-obese group. 

The prevalence of osteoporosis at either site (either femoral neck or lumbar spine) was found to be 52% in normal weight postmenopausal women, 43.2% in those with obesity, and 50.4% in those with morbid obesity. 

There was a statistically significant difference in the BMD at the total hip and TBS (lumbar spine) between the groups, as determined by one-way ANOVA (Table 1). Tukey’s post hoc test revealed that the mean BMD (gm/cm^2^) at the femoral neck in women with morbid obesity was found to be significantly lower as compared to the age-matched postmenopausal obese controls (0.723 versus 0.762, *p*-value = 0.002). The bone microarchitecture was found to be significantly lower in those with morbid obesity (1.205) as compared to the other two groups (obesity 1.244.; non-obese 1.228) (*p* < 0.013). 

## 4. Discussion

The present study indicates that individuals with morbid obesity have a lower bone mineral density than those with obesity. The BMD at the femoral neck was found to be significantly lower in morbidly obese women as compared to the obese and non-obese age-matched subjects. Severe forms of obesity in postmenopausal Indian women may therefore predispose them toward fragility fractures, which may further limit their physical activity and promote weight gain.

A retrospective study from Spain on 832,775 postmenopausal women showed an increased risk of proximal fractures of the humerus in obese females compared to those normal or underweight (RR: 1.28, 95% CI: 1.04–1.58) [10]. A global longitudinal study of osteoporosis in women (GLOW) showed a higher risk of ankle and upper leg fractures but a lower incidence of wrist fractures in those obese [9]. Another large meta-analysis that included data from 25 prospective cohorts (398,610 women aged 20–105 years; mean age 63 years) concluded that, after adjustment for the BMD, the apparent protective effect of obesity on osteoporotic fractures is lost, and there is a greater increase in risk of leg and humerus fractures in morbidly obese women [15]. 

Though the bone mineral density at the femoral neck is significantly lower in the morbidly obese group, it also showed a trend at the lumbar spine, which may not have reached statistical significance due to the smaller numbers. In a previously published study in morbidly obese women who underwent bariatric surgery, it was found that, on follow-up, the femoral neck BMD declined much more than lumbar spine BMD [34]. Though no clear explanation is cited for this differential impact of obesity on the BMD, further studies are needed to explore the underlying mechanisms. The discordance about BMD and TBS at the lumbar spine has previously been reported in individuals with type 2 diabetes and obesity in several original articles and guidelines, including the ISCD-IOF guidelines and the most recently published ISBMR position statement [19,23,24,35]. Several mechanisms have been cited in the literature as causes for increased bone density despite poor bone architecture and high bone fragility in people with metabolic syndrome. These include hyperinsulinemia, increased adipose tissue, increased glycated end products, and a reduced bone turnover state [36]. In the current study, despite having 34% of individuals with dysglycemia among those with morbid obesity (which should be associated with a falsely elevated BMD), this group still had a significantly lower BMD as compared to the other groups. This implies that morbid obesity is associated with a significantly lower BMD despite the presence of diabetes in a third of the study subjects, and so, subjects with severe obesity should be evaluated irrespective of diabetes.

One of the proposed factors for lesser BMD in these individuals may be lower adiponectin and higher proinflammatory adipocytokines like TNF-α [37]. Some studies have suggested improvement in the BMD after starting infliximab, a TNF-α antagonist in patients with rheumatoid arthritis [38]. It is also postulated that there is less GH/IGF-1 in morbidly obese individuals, which is important for skeletal integrity [37].

Leptin, in addition to adipose tissue in the bone marrow, is also produced from osteoblastic cells and regulates appetite and weight gain. It also regulates osteoblast and osteoclast differentiation. Leptin also affects bone through its actions on the central nervous system—in particular, the hypothalamus. Adiponectin circulates in much higher concentrations than other adipocyte products. It regulates the metabolic and inflammatory pathways and appears to exert a negative effect on the bone mass. Adiponectin receptors have been identified in both osteoblasts and osteoclasts. It has also been proposed that adiponectin can activate transcription factor NF-κB, regulating proinflammatory gene expression according to its oligomerization state. Resistin has rarely been found to be expressed by adipocytes but is expressed by bone marrow and peripheral mononuclear cells. It acts as an inflammatory cytokine in the atherogenic process. A systematic review and meta-analysis by Biver et al. on the influence of adipokines on bone mineral density concluded that adiponectin is the most relevant adipokine (among leptin, resistin, and visfatin) negatively associated with BMD (pooled r from −0.14 to −0.4) independent of gender, fat mass parameters, and menopausal status [39].

There is also a high prevalence of Vitamin D deficiency and consequent secondary hyperparathyroidism in those morbidly obese, as described in multiple studies [40,41]. However, in our study, there was no significant difference between the three groups regarding the vitamin D levels. Morbidly obese individuals have higher bone resorption markers compared to normal and overweight individuals [37,41].

The individuals with morbid obesity had a significantly poor bone microarchitecture as compared to those with obesity or normal BMI. In another study by Povoroznyuk et al., the TBS was negatively associated with an increasing BMI [42]. The TBS has also been found to be a useful tool to assess the bone microarchitecture in patients undergoing bariatric surgery [43]. However, in view of the ethnic differences in the bone mineral parameters, it is important to assess the utility of the bone microarchitecture in different ethnicities [16,44].

Although this study did not look into the incidences of fractures in the three subgroups, the two largest retrospective meta-analyses showed higher incidences of fractures in morbidly obese postmenopausal women [9,10]. 

Though conventionally due to the increased mechanical load, the increased bioavailability of estrogens and increased secretion of insulin from β cells are thought to have a bone protective effect [45]. The increased propensity of falls, physical inactivity, increased trochanteric soft tissue thickness, sarcopenic obesity, and comorbid illnesses may counteract the protective effect of fat and result in poor bone strength in morbidly obese women [46].

Furthermore, obesity per se may be associated with comorbidities like chronic obstructive lung disease, diabetes mellitus, increased parathormone levels, vitamin D deficiency, and functional hypercortisolism. Obese individuals are more likely to have diabetes, which itself may predispose them to develop fractures. However, irrespective of the poor bone health in individuals with diabetes, they have a falsely high bone mineral density [47]. In our study, we found morbidly obese individuals to have a lower mean BMD despite having a larger number of people with diabetes, further emphasizing that these individuals warrant screening, as, despite having more individuals with diabetes, their BMD was found to be lower. Morbidly obese women may have low Vitamin D, secondary hyperparathyroidism, high bone resorption, decreased GH/IGF-1, and high proinflammatory adipocytokines as the key molecular factors responsible for low BMD in these individuals.

This is the first Indian study to evaluate the bone mineral density and bone microarchitecture in a cohort of morbidly obese postmenopausal women. The limitations of this study include that the data on women with morbid obesity is from a tertiary care hospital and may not represent the problem statement in the community. In addition, details regarding the bone resorption markers, bone formation markers, and physical activity were not collected in this study. To conclude, based on this pilot study, we propose that postmenopausal women with morbid obesity may have a negative association of body mass index with bone mineral density. Further studies will be needed to validate this research finding and to explore the prevalence and mortality associated with incident fractures prevalent in people with morbid obesity. A study with a larger sample size would also enable studying the impact of individual comorbidities on bone health in people with morbid obesity. Moreover, since the BMI is not the best marker for an obesity assessment, especially in the South Asian region [26,30,32], the relationship of other obesity indicators (waist−height ratio, visceral adiposity assessment, body fat percentage, lean mass, etc.) with bone health should be studied in future cohorts.

## Figures and Tables

**Table 1:** The demographic details and bone mineral parameters in the three groups.

3 Study Groups →	Group—1 Non Obese (BMI ≤ 25 kg/m^2^) *N* = 85	Group—2 Obese (BMI ≥ 25–35 kg/m^2^) *N* = 80	Group—3 Morbidly Obese (BMI ≥ 35 kg/m^2^) *N* = 85	*p*-Value
Mean(SD) of the Parameter Studied ↓				
Age (in years)	58.1 (4.4)	57.6 (5.1)	58.1 (6.3)	0.787
BMI (kg/m^2^)	21.1 (2.3)	27.2 (3.9)	38.5 (3.2)	<0.001
Albumin corrected calcium (mg/dL)	8.78 (0.46)	8.84 (0.5)	8.92 (0.06)	0.067
Serum phosphorus (mg/dL)	3.6 (0.4)	3.5 (0.6)	3.4 (0.9)	0.150
25-OH Vitamin D (ng/mL)	26.8 (8.7)	24.5 (11.3)	23.2 (12.6)	0.098
BMD at Femoral Neck (g/cm^2^)	0.756 (0.104)	0.762 (0.120)	0.723 (0.106)	0.002
BMD at Lumbar Spine (g/cm^2^)	0.825 (0.101)	0.875 (0.153)	0.866 (0.113)	0.051
BMD at Total Hip (g/cm^2^)	0.833 (0.113)	0.800 (0.141)	0.860 (0.124)	0.070
Trabecular bone score at Lumbar Spine	1.228 (0.050)	1.244 (0.090)	1.205 (0.105)	0.013

## Data Availability

The data presented in this study are available on request from the corresponding author. The data are not publicly available due to sensitive nature of data of patients from the clinic.
